# Mixed-Reality-Assisted Puncture of the Common Femoral Artery in a Phantom Model

**DOI:** 10.3390/jimaging8020047

**Published:** 2022-02-16

**Authors:** Christian Uhl, Johannes Hatzl, Katrin Meisenbacher, Lea Zimmer, Niklas Hartmann, Dittmar Böckler

**Affiliations:** Department of Vascular and Endovascular Surgery, University Hospital Heidelberg, Im Neuenheimer Feld 420, 69120 Heidelberg, Germany; christian.uhl@med.uni-heidelberg.de (C.U.); katrin.meisenbacher@med.uni-heidelberg.de (K.M.); lea.zimmer@gmail.com (L.Z.); niklas.hartmann@med.uni-heidelberg.de (N.H.); dittmar.boeckler@med.uni-heidelberg.de (D.B.)

**Keywords:** mixed reality, virtual reality, vascular surgery, vascular access, femoral artery, endovascular

## Abstract

Percutaneous femoral arterial access is daily practice in a variety of medical specialties and enables physicians worldwide to perform endovascular interventions. The reported incidence of percutaneous femoral arterial access complications is 3–18% and often results from suboptimal puncture location due to insufficient visualization of the target vessel. The purpose of this proof-of-concept study was to evaluate the feasibility and the positional error of a mixed-reality (MR)-assisted puncture of the common femoral artery in a phantom model using a commercially available navigation system. In total, 15 MR-assisted punctures were performed. Cone-beam computed tomography angiography (CTA) was used following each puncture to allow quantification of positional error of needle placements in the axial and sagittal planes. Technical success was achieved in 14/15 cases (93.3%) with a median axial positional error of 1.0 mm (IQR 1.3) and a median sagittal positional error of 1.1 mm (IQR 1.6). The median duration of the registration process and needle insertion was 2 min (IQR 1.0). MR-assisted puncture of the common femoral artery is feasible with acceptable positional errors in a phantom model. Future studies should aim to measure and reduce the positional error resulting from MR registration.

## 1. Introduction

Percutaneous puncture of the common femoral artery (CFA) is a frequently used technique to gain arterial vascular access and is applied in a variety of medical specialties such as vascular surgery, cardiology, interventional radiology, and neuroradiology. The technique of femoral arterial puncture has changed in recent decades. Initially the optimal puncture site was determined by palpation of the pulse and the location of the CFA relative to the femoral head in projection radiography. In recent years the use of sonography with real-time two-dimensional visualization of the target vessel has become the gold standard. Although the use of sonography has reduced the complication rate after femoral arterial access, there are still relevant puncture-associated complications in 3–18% of cases today [1,2]. Complications during vascular arterial access mainly arise from suboptimal localization of the puncture site, in addition to the quality of the targeted vessel (diameter, calcification). Inadvertent puncture of the superficial femoral or profound femoral arteries can result in false aneurysms, local dissections with subsequent lower limb ischemia or bleeding complications [2,3]. Puncture of the external iliac artery proximal to the femoral ligament can result in catastrophic retroperitoneal hemorrhage, which is associated with high morbidity and mortality [4,5,6]. Arterial puncture of severely calcified vessel areas can lead to local vascular injury and failure of vascular closure devices, which often leads to open surgical conversion to control resulting bleeding complications. In summary, optimal visualization of vascular anatomy is crucial to perform safe femoral arterial access [3,7].

**Figure 1:**
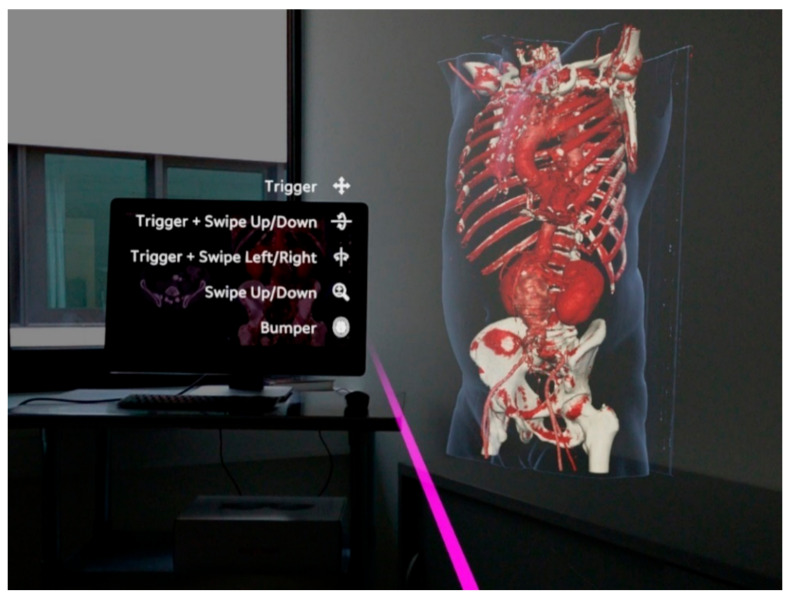
Observer’s point of view using the Magic Leap 1 (Plantation, Magic Leap, FL, USA) and the Elements Viewer software (Brainlab AG, Munich, Germany). The 3D virtual model on the right is based on a computed tomography angiography (CTA) of a patient with an abdominal aortic aneurysm. The virtual pink pointer is generated in front of the manual controller and can be used to interact with the virtual model and the user interface of the software.

In recent years, extended reality (XR) applications such as virtual reality (VR), augmented reality (AR) and mixed reality (MR) have been used more extensively in healthcare. MR is a technology that allows the projection of three-dimensional (3D) virtual models into the user’s field of view by utilizing a head-mounted display (HMD). Currently commercially available HMDs for MR are the Magic Leap 1 (Plantation, Magic Leap, FL, USA) and the Microsoft HoloLens (Microsoft, Redmond, DC, USA). Whereas in VR the user enters a completely simulated virtual environment, MR overlays the physical environment with digital content, offering the advantage for users to interact with these objects while still being able to see real objects. The use of MR in a medical context has previously been described in patient education, teaching and intraoperative navigation in a wide variety of surgical specialties such as orthopedics, neurosurgery, and plastic surgery [8,9,10,11,12,13,14,15,16,17,18,19,20]. Although the futuristic vision of MR as a navigational tool in surgery appears to be especially promising, there are currently many challenges remaining with regards to the technical feasibility and the accuracy of virtual model registration, in addition to the stabilization of the virtual model in a spatial environment. Different methods of registration have been investigated, ranging from manual registration and automatic registration using optical tracking markers to registration assisted by commercially available navigation systems. The most investigated clinical application in the past has been pedicle screw placement during spine surgery, which has demonstrated promising initial results in several pilot studies [21,22,23,24,25,26]. In the field of vascular surgery, initial studies have also been conducted to investigate the potential of MR [16,27,28]. MR can be utilized to display a three-dimensional reconstruction of a patient’s vascular anatomy, derived from computed tomography angiography (CTA, Figure 1). Femoral arterial access may potentially be a clinical application for MR by improving vessel visualization, allowing the surgeon to focus on performing vascular punctures without shifting attention to a secondary monitor, and thereby increasing patient safety during vascular access procedures. The aim of the present proof-of-concept study was to evaluate the feasibility and to quantify the positional error of a MR-assisted puncture of the CFA in a phantom model utilizing the Magic Leap 1 (Plantation, Magic Leap, FL, USA) and a conventional navigation platform (Curve^®^ navigation platform, Brainlab AG, Munich, Germany)

## 2. Materials and Methods

### 2.1. Vascular Phantom Model

**Figure 2:**
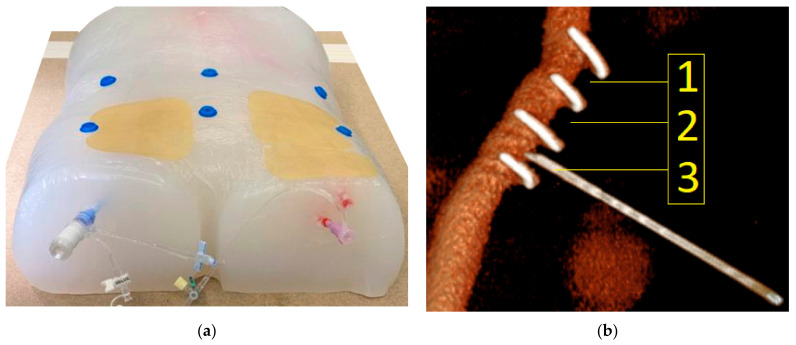
(**a**) Vascular phantom model. The silicone torso incorporated a full aorto-iliac vasculature that was filled with contrast medium via the two externalized sheaths. Six radiopaque markers were placed on the surface of the model. The area of interest in the groin region was covered with a replaceable plastic pad to blind the observer for the previous puncture attempts. (**b**) Corresponding three-dimensional reconstruction based on a CTA scan of the phantom model (Pie Medical Imaging BV, Maastricht, The Netherlands). The transversely oriented radiopaque copper markers are displayed, which defined the three target areas (1, 2 and 3). In the displayed example, the needle punctures target area 3.

The phantom model consisted of a silicone body (SF-33 silicone, around 16 kg, silikonfabrik.de, Ahrensburg, Germany) shaped like a human torso with incorporated sacrum, pelvis, and femoral joints, and proximal femoral bones and the two lowest lumbar vertebrae (3B Scientific GmbH, Dresden, Germany). The model featured a full vascular anatomy consisting of the abdominal aorta including visceral arteries, in addition to iliac arteries and a common femoral artery on the right side and a common, superficial, and profound femoral artery on the left side (Vascular international, Kerns, Switzerland). The model also had two vascular sheaths (22 French Dry-Seal sheath, Cook Medical, Inc., Bloomington, IN, USA and a 10 French sheath, Terumo, Tokyo, Japan) on the right and left side, respectively. The model also had radiopaque markers made of copper in defined distances as borders of predefined target areas (target areas 1, 2 and 3). Each target area measured 0.8 cm × 1.0 cm (Figure 2b). The target area was localized at a depth of 1.5 cm beneath the surface of the phantom model. The vasculature of the phantom model was filled with contrast medium via the sheaths. The model was prepped with six radiopaque markers on its surface to allow navigation. A baseline CTA scan was performed on the model, which demonstrated adequate differentiation of modeled bone, vessel, and soft tissue structures, in addition to good visualization of the integrated radiopaque copper markers attached to the vasculature. To allow multiple punctures of the same target area, the vasculature was prepped with a plastic cover on the targeted areas.

### 2.2. Experimental Set-Up

**Figure 3:**
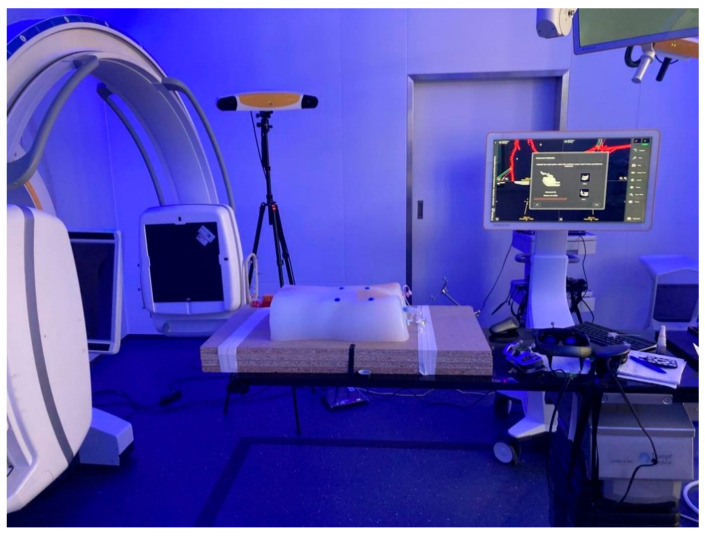
Experimental set-up: Phantom model (center), X-ray device (Siemens Cios alpha C-arm, Siemens Healthcare GmbH, Erlangen, Germany) for cone-beam CTA (left), Curve^®^ Navigation system (Brainlab AG, Munich, Germany) for optical tracking with tripod camera system and workstation (background), and HMD on the O.R. table (right).

For acquisition of pre-procedural CTA images, an AIRO 32 slices CT scanner (Brainlab AG, Munich, Germany) was used. The experimental setup consisted of the Curve^®^ navigation platform (Brainlab AG, Munich, Germany) running both the Cranial navigation software (Brainlab AG, Munich, Germany) and a software prototype (Brainlab AG, Munich, Germany), allowing for a MR-assisted presentation of the navigation. The latter was conducted using the Magic Leap 1 (Magic Leap 1, Plantation, Magic Leap, FL, USA) as HMD. An 18-gauge needle with an optical tracking reference array attached to it was used to perform punctures of the phantom model. Post-procedural verification scans were performed using a Siemens Cios alpha C-arm (Siemens Healthcare GmbH, Erlangen, Germany). The experimental set-up is displayed in Figure 3.

### 2.3. Workflow

**Figure 4:**
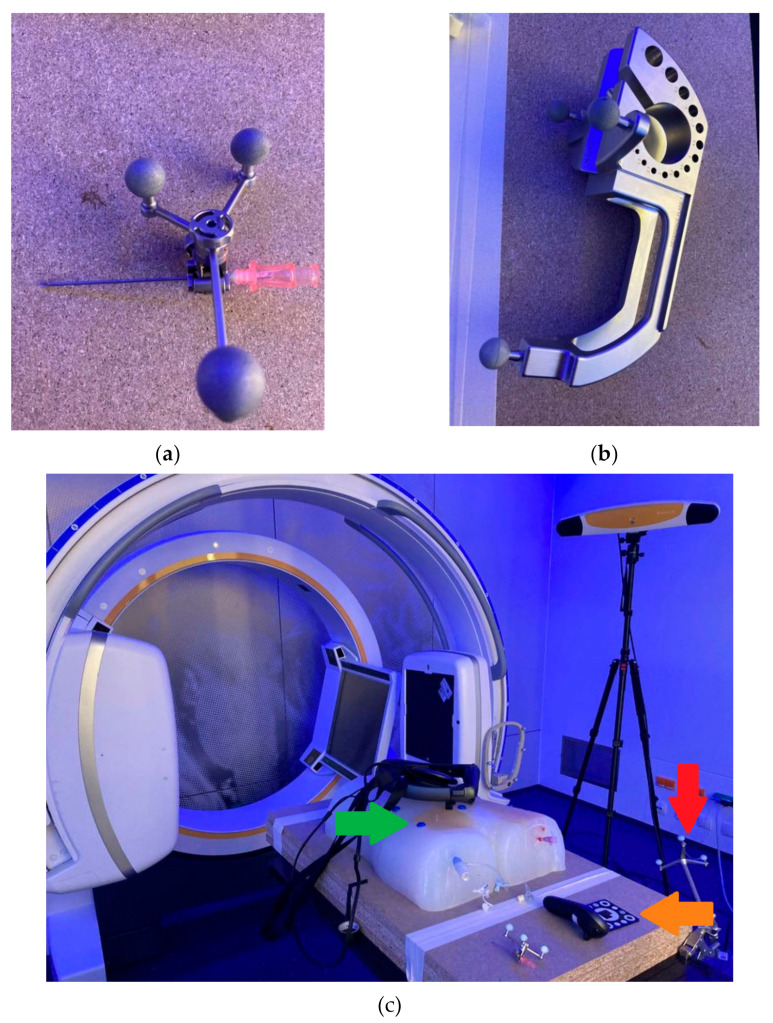
(**a**) Eighteen-gauge needle that was used for vascular punctures with an optical reference array attached to it. (**b**) Calibration matrix used to allow the navigation system to track the position of the needle. (**c**) Red arrow: optical tracking reference array attached to the O.R. table to allow the navigation system to co-register the CT dataset with the phantom model. Green arrow: One of six radiopaque markers that allow registration of the CT dataset with the phantom model using the navigation system using the paired-point registration method. Orange arrow: Custom disposable hybrid marker (Brainlab AG, Munich, Germany) that was placed in the field of view of the Magic Leap 1 and the camera of the navigation system to align both coordinate systems, which allowed for an automatic alignment of the virtual 3D model to the phantom.

First, six radiopaque donut-shaped markers were placed evenly distributed on the surface of the phantom model (Figure 2a). Next, as a basis for the creation of the 3D virtual model, a CT scan of the phantom was performed. The phantom was then placed on an operating room (O.R.) table with an optical tracking reference array attached to it near the phantom model (Figure 4c). Finally, the navigation system was set up next to the O.R. table with the phantom being positioned within the field-of-view-of the navigation system’s optical tracking camera. The CT dataset was then loaded to the navigation system.

For the co-registration of the CT dataset to the phantom, the paired-point registration method was used. In this case, the radiopaque markers were defined as reference landmarks (center of marker defined as registration target) in the navigation software and then acquired on the phantom using a tracked pointer. The registration was verified by placing the tracked pointer on different landmarks on the phantom and comparing its position to the position indicated on the images of the navigation system.

Having registered the CT dataset to the phantom, a trajectory was defined in the image dataset, serving as a reference line to the target region in the phantom.

**Figure 5:**
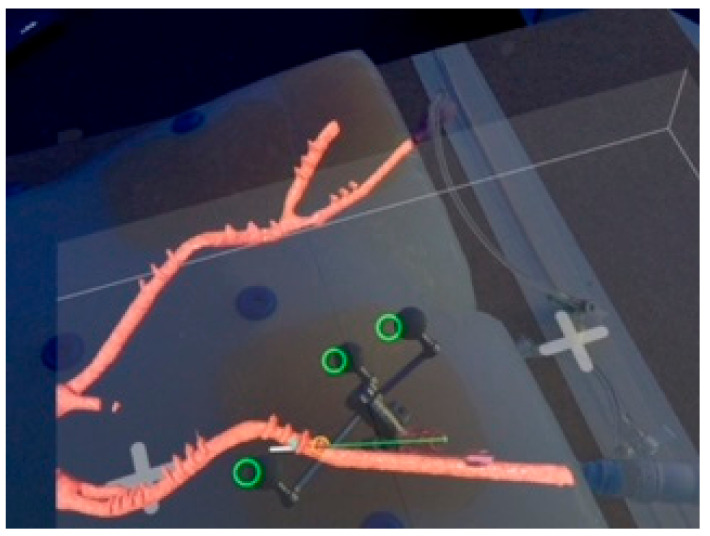
Observer’s point of view during puncture of the right common femoral artery of the phantom model. The observer can see the phantom model with the registered 3D virtual model of the iliaco-femoral vasculature (red) prepped with radiopaque copper markers. The green line represents the planned trajectory of the puncture. The white line represents the digital projection of the needle. The green rings demonstrate the registration between the physical and virtual needle in the MR environment. The offset between the virtual rings and the reference markers is due to the position of the camera in the Magic Leap headset, which is off-center.

Next, an optical tracking reference array was attached to the needle, which was registered and calibrated using a calibration matrix (Figure 4a,b) allowing the navigation system to track the position of the needle. The user then connected the Magic Leap 1 headset to the navigation software prototype. Subsequently, a custom disposable hybrid marker (Figure 4c) (Brainlab AG, Munich, Germany) was placed in the field of view of the Magic Leap 1 and the camera of the navigation system to align both coordinate systems. This step allowed for an accurate automatic alignment of the virtual 3D model to the phantom. The alignment of the 3D model with the phantom was confirmed visually by checking the correlation of the virtual green circles and the marker spheres of the reference array (Figure 5).

To place the tracked needle in the target area of the right CFA, the user aligned the virtual representation of the needle with the planned trajectory visualized in the 3D model (Figure 5). Overall, a total of fifteen punctures were performed. Following each puncture, a cone-beam CTA was performed to visualize the physical needle position within the phantom model. 

### 2.4. Measurement of Positional Error

Each scan was evaluated for positional error of needle placement using center line reconstructions with dedicated software (3mensio Medical Imaging BV, Pie Medical Imaging BV, Maastricht, The Netherlands). The experimental set-up is presented in Figure 3. The observer’s point of view when performing CFA puncture is presented in Figure 5. 

**Figure 6:**
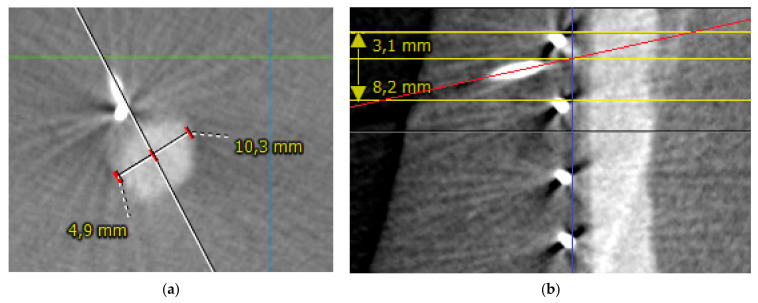
(**a**) Measurement of positional error in the axial plane in puncture number 5. The white line represents the path of the needle towards the vessel. The vessel diameter is measured as 10.3 mm. The distance from intersection of the needle path with the perpendicular vessel diameter to the lateral border of the vessel is 4.9 mm. The middle of the axial vessel diameter is at 5.15 mm. Therefore, positional error is 0.25 mm. (**b**) Measurement of positional error in sagittal plane in puncture number 7. The red line represents the path of the needle. The yellow lines represent the copper markers. The blue line represents the vessel surface. The intersection of the red and blue lines marks the puncture site. The distance from the upper copper marker to the puncture site is measured as 3.1 mm with the center of the target area as 4.1 mm, which translates to a sagittal positional error of 1 mm.

Positional error was measured in axial and sagittal planes. A centerline reconstruction was performed from every post-puncture cone-beam CTA scan. Positional error was measured in axial planes by measuring the distance from the intersection of the needle path with the perpendicular vessel diameter to the lateral border of the vessel. The difference between half of the vessel diameter and the measured diameter from the lateral border to the intersection of the needle path is defined as axial positional error (Figure 6a).

Sagittal positional error was measured as the difference between the distance of the upper copper marker limiting the respective target area to the intersection of the needle path with the vessel surface and half of the distance between the two copper markers defining the respective target area (Figure 6b).

Measurements were performed using 3mensio Medical Imaging BV software (Pie Medical Imaging BV, Maastricht, The Netherlands). Technical success of the puncture was defined as puncture in the targeted area (1, 2 or 3) and puncture of the anterior circumference of the vessel. 

### 2.5. Statistical Analysis

Medians with interquartile range (IQR) are reported. Statistical analysis was performed using R-statistics [29]. 

## 3. Results

### 3.1. Phantom-3D Virtual Model-Registration 

The registration of the phantom model with the virtual model as well as subsequent display in the MR environment was achieved in 15/15 cases (100%). After the experimental set-up was completed, the median duration of the registration process including needle calibration and the performance of arterial puncture was 2 min (IQR 1). 

### 3.2. Positional Error

**Figure 7:**
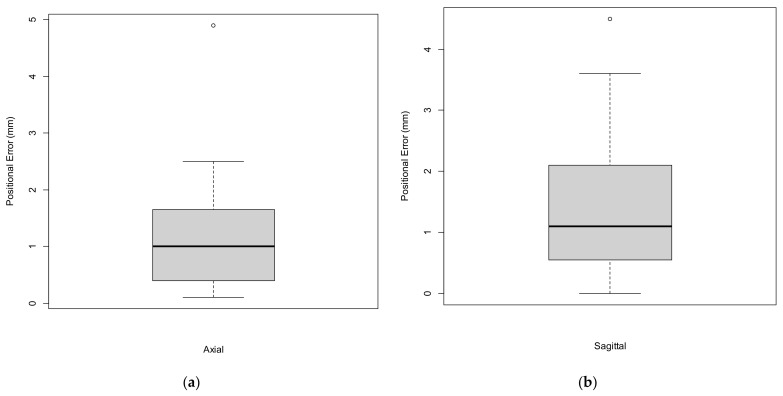
Boxplots representing the axial (**a**) and sagittal (**b**) positional errors.

In total, there were 15 attempts of right CFA puncture. The median axial positional error was 1.0 mm (IQR 1.3) with a median sagittal positional error of 1.1 mm (IQR 1.6). Positional error is presented in Figure 7a,b and Table 1. The ventral circumference of the vessel was punctured in 14/15 cases (93.3%). In all 14 cases, the mid-portion, defined as the central 5 mm of the anterior circumference, was punctured. In the remaining case the vessel was punctured at the lateral edge of the vessel besides the anterior circumference and was therefore counted as a technical failure. In 10/15 cases (66.6%), the mid-portion, defined as the central 4 mm of the distance between two copper markers in the sagittal plane, was punctured. Overall, following the above-mentioned definition, technical success of the CFA puncture was achieved in 93.3% of cases.

## 4. Discussion

The introduction of sonography has led to a significant reduction in complications arising from femoral arterial access. Nevertheless, femoral arterial access is associated with complications in 3–18% of cases [1,2,30]. Considering the overall number of patients undergoing femoral arterial access each year in a broad variety of medical specialties, this represents a substantial number of affected patients. Adequate visualization of the target vessel is key for a safe vascular access procedure. Previous studies have already suggested MR-assisted vascular access procedures [16,31]. The study of Groves et al. suggested improved accuracy results with the use of an HMD when compared with traditional sonography-guidance during central venous catheter placement in a phantom study [16]. To the best of our knowledge, the present study is the first to investigate the feasibility and the positional error of a MR-assisted approach for CFA access using an HMD combined with a commercially available navigation system. 

Although currently commercially available navigational systems (without MR) have demonstrated positional errors of ≤2 mm, the error resulting from registration with the MR environment has yet to be investigated [32]. In the present study, the cumulative positional error between initial imaging registration in the navigational system and the actual MR-assisted puncture of the femoral artery, including the human error during the puncture itself, was in line with previously reported accuracies in spine surgery with a similar experimental set-up using an HMD and a commercially available navigation system [22,23,33]. Although the quantified positional error was generally sufficient for CFA access in a phantom model, with 14/15 technically successful punctures (93.3%), there was one failed puncture (6.7%) and 1/3 of punctures were outside the mid portion of the targeted area. Considering that CFA puncture was performed repetitively on an immobile phantom model and in an experimental setting using an advanced navigation system, the results of this study demonstrate the necessity of further improvements in positional accuracy of the registration process before transfer of this technology into clinical practice can be suggested. Previous studies have demonstrated that the number, size, and position of the radiopaque markers may play an important role for accurate pattern recognition [34]. Other factors, such as calibration errors, are also known to have an influence on positional error and these sources of error should be subject to systematic investigation in the future [34,35]. Furthermore, not only could technological improvements advance the applicability of MR technology in surgical guidance, but also the use of creative synergies of existing technology. As an example, the combination of MR with sonography for femoral arterial access could serve as an interesting clinical application. The advantages of real-time needle visualization and its interaction with the target vessel combined with the excellent topographic display of a high-quality 3D virtual model, including vessel calcifications, naturally incorporated into the observer’s field of view, may harbor the potential to further increase the safety of patients undergoing femoral arterial access. 

A notable limitation of the study is that there was no control group performing sonography-guided puncture of the CFA to allow the comparison of technical success rates and positional errors with the navigated MR-technique, because the silicone that was used to produce the phantom model could not be investigated with sonography due to air entrapments within the material. Additionally, the silicone limited the needle movement in the modeled subcutaneous tissue due to its firmness. The phantom model also had a very favorable anatomy for CFA access with a target area that was incorporated rather superficially within the phantom model. Furthermore, all punctures were performed repetitively by the same observer, which could have led to a learning curve and/or fatigue effect. Therefore, results should not be prematurely generalized. Another limitation of the study is that the measured cumulative positional error cannot be exactly differentiated between the different sources of error. Although the error of the navigational system can be assumed to be rather low, because it has already been validated for accuracy, the proportion of human error and the error resulting from MR registration remains uncertain. Hence, conclusions with regards to the actual registration accuracy of the applied MR registration method need to be made cautiously. Measuring registration error of MR-assisted navigation systems is challenging, and previous studies, in addition to the present study, have focused on measuring the positional errors with which a user can perform a defined clinical task [8,9,12,19]. In the present study, positional error was quantified according to needle positioning in post-puncture cone-beam CTA. Considering accumulation of the above-mentioned limitations, the quantified positional errors resulting from this proof-of-concept study need to be interpreted cautiously. For the future, MR-assisted navigation should not be reliant on advanced navigation systems with the associated technical and infrastructural requirements in order to increase its clinical feasibility. Therefore, alternative methods of image registration should be investigated. The development of more realistic models in different variants to allow the precise investigation of new technologies such as MR may also be of interest for future efforts to systematically study the phenomenon of registration errors in different clinical applications.

MR is a promising and futuristic imaging viewing technology and several studies have investigated its application in patient and student education, and procedural planning [36,37,38,39,40]. However, the true potential of MR can only be utilized in an application in which the physical and the virtual objects are equally obligatory components of the application. At this time, applications such as these have been reported scarcely but increasingly in the literature. We have demonstrated such an application in the field of vascular medicine. 

## 5. Conclusions

MR-assisted puncture of the common femoral artery is feasible with acceptable positional errors in an experimental setting using an advanced navigation system in a phantom model. Future studies should aim to measure and reduce the positional error resulting from MR registration. 

## Figures and Tables

**Table 1:** Axial and sagittal positional errors (*N* = 15).

Puncture ID	Positional Error (Axial) (mm)	Positional Error (Sagittal) (mm)	Target Zone	Technical Success
1	2.1	0.6	1	Yes
2	2.45	2.55	2	Yes
3	0.1	1.45	3	Yes
4	0.95	0.5	1	Yes
5	0.25	2	2	Yes
6	0.5	2.05	3	Yes
7	0.65	1	1	Yes
8	1.2	2.05	2	Yes
9	0.85	0.5	3	Yes
10	0.1	1.05	1	Yes
11	0.25	0.55	2	Yes
12	1.05	0.05	3	Yes
13	1.45	0	1	Yes
14	1.8	4.5	2	Yes
15	4.85	3.55	3	No

## Data Availability

The data reported in this study are available upon requested from the corresponding author.
